# The molecular and functional characterization of ferritins in the hard tick *Hyalomma rufipes*

**DOI:** 10.1186/s13071-022-05515-0

**Published:** 2022-10-14

**Authors:** Zhihua Gao, Peijing Zheng, Kuang Wang, Xin Ji, Yanqing Shi, Xuecheng Song, Jingze Liu, Zhijun Yu, Xiaolong Yang

**Affiliations:** Hebei Key Laboratory of Animal Physiology, Biochemistry and Molecular Biology, Hebei Collaborative Innovation Center for Eco-Environment, Ministry of Education Key Laboratory of Molecular and Cellular Biology, College of Life Sciences, Hebei Normal University, Shijiazhuang, 050016 China

**Keywords:** *Hyalomma rufipes*, Ferritin, Function assessment, Blood-feeding, Midgut

## Abstract

**Background:**

The protein ferritin, which plays an important role in the maintenance of iron homeostasis, is indispensable for iron detoxification, resistance to oxidative stress and innate immunity. Ticks, which are obligate blood-sucking ectoparasites, have to deal with a large amount of iron when they take a blood meal.

**Methods:**

Sequence analysis was undertaken using bioinformatics. A recombinant (r) expression vector, rferritin, was constructed for a prokaryotic expression system. A quantitative polymerase chain reaction platform was used to detect the spatial and temporal expression patterns of target genes and their responses to a low temperature environment. Knockdown of the ferritin genes through RNA interference was used to analyze their effects on physiological parameters of ticks.

**Results:**

Two ferritin genes, *HrFer1* and *HrFer2*, were cloned from the tick *Hyalomma rufipes*. Their open reading frames are 519 base pairs (bp) and 573 bp in length, and number of coding amino acids 170 and 190, respectively. The phylogenetic tree showed that *HrFer1* and *HrFer2* have a close evolutionary relationship with the H subunit of ferritin. In vitro experiments showed that rHrFer1 and rHrFer2 had concentration-dependent iron chelating activity. The relative expression of the two ferritin genes was higher in the ovary and midgut of *H. rufipes*. RNA interference results demonstrated that *HrFer1* and *HrFer2* expression had a significant effect on engorged body weight, number of eggs laid, and mortality of *H. rufipes*, and that *HrFer2* also had a significant effect on feeding duration. Furthermore, the relative expression of ferritin decreased significantly in a low temperature environment, suggesting that *HrFer1* and *HrFer2* play a regulatory role in the cold stress response of *H. rufipes*.

**Conclusions:**

The results of the present study improve our understanding of the involvement of ferritins in tick blood-feeding.

**Graphical Abstract:**

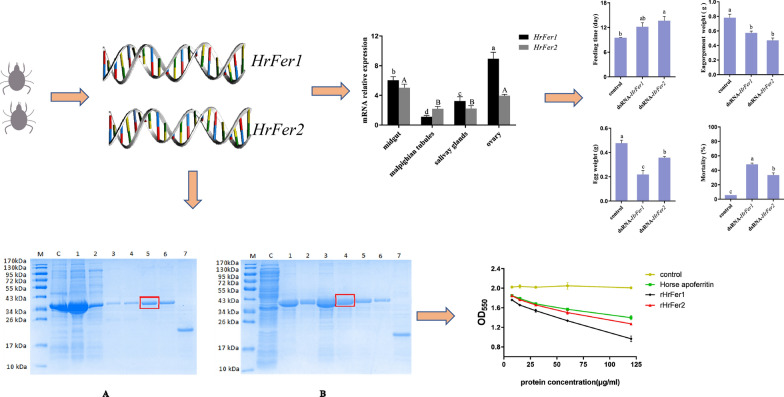

## Introduction

As obligate blood-sucking ectoparasites, ticks acquire essential nutrients solely from the ingested blood of their host for their development and reproduction [1]. During feeding, the host red blood cells, which contain a large amount of iron, enter the tick body [2]. The iron and hemoglobin ingested by ticks reach the cells of various tissues and organs in which ferritin is stored; ferritin plays a role in iron detoxification whereby excess iron is regulated to maintain it at a physiological level [3]. A variety of microorganisms may also be ingested from the host into the tick intestine during blood-feeding [4]. Hence, it is considered very important to understand the functions of the ferritin digested from a tick blood meal in intestinal immunity, as this might shed light on the development of immunological resilience and be of use for future tick control.

In China, the tick *Hyalomma rufipes* is mainly distributed in Inner Mongolia, Shanxi, Ningxia and Xinjiang, and parasitizes mammals such as sheep, camels, cattle and horses, and occasionally humans. It can spread *Babesia* spp., *Rickettsia* spp., Crimean-Congo hemorrhagic fever virus, human typhus fever virus and *Babesia occultans*, which have serious consequences for animal husbandry and human health [5]. The two-host life cycle of *H. rufipes* is completed, on average, in 179 days, with the immature ticks (larva and nymph) feeding for about 15-28 days, and the adults feeding for 12-19 days (e.g. on rabbits).

Ticks have evolved complex physiological and biochemical mechanisms to deal with their exposure to low environmental temperatures. They are able to persist in cold environments through the accumulation in their cells of small molecules that play a role in biochemical mechanisms that protect them against low temperatures. Preliminary laboratory work showed that, after long-term cold acclimation, these molecules significantly increased in nymph and adult *Haemaphysalis longicornis*, and that their supercooling capacity was significantly enhanced. Dynamic changes in lipids, antifreeze proteins and small molecular sugars are the main strategies for improving cold resistance in ticks. It has also been shown that ferritin in ticks has functions related to their cold resistance.

In this study, we characterized two ferritin genes of *H. rufipes* and elucidated their molecular phylogeny and transcription profiles in different tick tissues. We evaluated in vitro the iron deprivation activity of prokaryotic recombinant ferritins, and explored in vivo their pleiotropic functions in blood meal digestion and cold responses of *H. rufipes*. The results presented here shed light on the important roles of ferritins in the development of ticks.

## Methods

### Collection and rearing of ticks

Unfed *H. rufipes* adults were collected by flagging and dragging vegetation in Wuhai county, Inner Mongolia Autonomous Region, northern China. Ticks were transported to the laboratory and fed on the blood of rabbits. Briefly, the larvae and adults were released into cloth bags glued onto the ears of domestic rabbits *Oryctolagus cuniculus domestica* for feeding. Rabbits were maintained in a room at 50% relative humidity and 25–27 °C and were exposed to daylight. After engorgement and detachment, the ticks were collected, placed in glass tubes containing one folded filter paper, and stored in an incubator (75 ± 5% relative humidity, 6:18 h light:dark cycle, 26 ± 1 °C). All the experiments in this study were conducted with the approval of the Animal Ethics Committee of Hebei Normal University (protocol number IACUC-157036).

### Total RNA extraction and complementary DNA synthesis

Total RNA was extracted from unfed adults using Trizol reagent (Takara, Dalian, China) in accordance with the manufacture’s protocol, and the quality of the extracted RNA was assessed using electrophoresis on a 1% agarose gel coupled with spectrophotometry. PrimeScript One Step Reverse Transcription-PCR version 2 (Takara) was used to synthesize the first-strand complementary DNA (cDNA), in accordance with the manufacturer's instructions. The cDNA was then stored at −80 °C until use.

### Cloning and sequence analysis

Specific primers for *HrFer1* and *HrFer2* (*HrFer1*, forward primer 5′-ATGGCCGCTACTCAGCC-3′, reverse primer 5′-TCAGTCGGACAGGGTCTCC-3′; *HrFer2*, forward primer 5′-ATGTTTCGAATTGTAGTGCT-3′, reverse primer 5′-AAGCAAACACTAGGTGCG-3′) were designed using Primer Premier 5.0 software (Premier Biosoft International, Palo Alto, CA) and used to amplify the corresponding open reading frame (ORF). The polymerase chain reaction (PCR) conditions were as follows: 94 °C for 4 min, followed by 35 cycles each at 94 °C for 30 s; 57 °C for 30 s; 72 °C for 1 min; and a final elongation at 72 °C for 10 min. The PCR products were resolved by agarose gel electrophoresis (1%), and the DNA bands of expected size were cut and purified using DNA Gel Extraction Kit (Axygen, Shanghai, China) in accordance with the manufacturer’s instructions. The purified PCR products were inserted into T-Vector pMD19 for sequencing by Tongyong (Chuzhou, China). The confirmed cDNAs of *HrFer1* and *HrFer2* were blasted [National Center for Biotechnology Information (NCBI) (http://blast.ncbi.nlm.nih.gov/Blast.cgi)], and the complete sequences of *HrFer1* and *HrFer2* deposited in the NCBI Nucleotide database under the respective accession numbers UTH39182 and UTH39183. The online tool ExPASy (http://web.expasy.org/computepi/) was used to predict their molecular weight and theoretical isoelectric point. The iron-responsive elements were predicted using web servers for RNA secondary structure prediction (http://rna.urmc.rochester.edu/RNAstructureWeb/Servers/Predict1/Predict1.html). The conserved domains were predicted using the Expasy PROSITE database (http://prosite.expasy.org). Multiple sequence alignment was conducted using CLUSTALW 2.1

Sequence alignment and phylogenetic analysis were performed for *HrFer1* and *HrFer2* and additional nucleotide sequences of ferritins from other species were retrieved from GenBank. The phylogenetic tree was constructed using MEGA 5.10 with the maximum likelihood method (1000 times) with nearest-neighbor interchange and the bootstrap test for phylogeny [6].

### Transcription profiles of *HrFer1* and *HrFer2*

To quantify the expression of *HrFer1* and *HrFer2* in the ticks, total RNA was prepared from different tissues (midgut, salivary glands, ovaries and Malpighian tubules) of feeding females 10 days after mating. The expression of *HrFer1* and *HrFer2* was evaluated using quantitative (real-time) PCR (qPCR). Briefly, the first strand cDNA was synthesized using Easy Script first-strand cDNA Synthesis SuperMix kit (TransGen, Beijing, China) in accordance with the manufacturer’s protocol. Fifty microliters of standard PCR reaction mixture was amplified with 1 μL of the above products and gene-specific primers (HrF1, forward primer 5′-GTCACAGGATGAATGGGGC-3′, reverse primer 5′–AGAAAGCTCTTTGATTGCCT-3′; HrF2, forward primer 5′-GCACATCTCGCCAACAACA-3′, reverse primer 5′-GGACGCTCATCCAGGTCG-3′); samples were also amplified using β-actin (accession number AY254898) primers (forward primer 5′-CGTTCCTGGGTATGGAATCG-3′, reverse primer 5′-TCCACGTCGCACTTCATGAT-3′) as the control. Real time (RT) qPCR (RT–qPCR) was performed using TransStart Tip Green qPCR SuperMix (TransGen).

The adults (10 ticks in each group) were exposed to a series of low temperatures (2 ℃, 4 ℃, 8 ℃, 16 ℃) and acclimated for 72 h; ticks incubated at 26 °C served as the control group [7]. Total RNA was extracted from the whole tick, and the relative expression of *HrFer1* and *HrFer2* quantified by qPCR as described above. Each analysis was carried out at least in triplicate.

### Prokaryotic expression system and protein purification

A prokaryotic expression system was used to obtain recombinant proteins of rHrFer1 and rHrFer2. Briefly, fragments from *HrFer1* and *HrFer2* were separately cloned into the pET-32(a+) vector (Takara), which is characterized by EcoRI and NotI restriction sites, and then transformed into competent *Escherichia coli* (Transetta DE3) cells (AxyGen, Shanghai, China) and cultured at 37 °C. Then isopropyl-β-d-thiogalactoside (1 mM) was added and the mixture incubated for 5 h to induce expression. The bacterial cells were centrifuged at 5500 *g* for 10 min at 4 °C, followed by two washes with phosphate-buffered saline (pH 7.4). Binding buffer (20 mM Tris-HCl, 500 mM NaCl, 5 mM imidazole, pH 7.9) was added to resuspend the bacterial cells, and the mixture was then sonicated in an ice bath, followed by centrifugation for 10 min at 12,000* g* and 4 °C. The resultant recombinant proteins were confirmed by 12% sodium dodecyl sulfate–polyacrylamide gel electrophoresis (SDS-PAGE). The recombinant proteins identified by SDS-PAGE were cut out and digested by enzyme hydrolysis, and analyzed by liquid chromatography–tandem mass spectrometry. Further purification was carried out using the QIAexpress Ni-NTA Fast Start Kit (Qiagen, Frankfurt, Germany) in accordance with the provided instructions, followed by dialysis to improve the purity of the rHrFer1 and rHrFer2 for further assays. Ultrafiltration was performed using Millipore ultrafiltration centrifuge tubes (Merck, Germany) with interception apertures of 10kDa and 50kDa, respectively, and centrifugation at 4000 *g* for 30 min. The BCA Protein Assay Kit (TransGen) was used to determine the concentration of the purified protein.

### Iron deprivation activity of rHrFer1 and rHrFer2

The iron deprivation activity of rHrFer1 and rHrFer2 was examined following the method of De Zoysa and Lee [8], with some modification. Briefly, the recombinant proteins were diluted to 1 mL in a serial dilution, then 20 μL of 2 mM FeCl_2_ was added to each and the mixtures incubated at room temperature (~22 ℃) for 10 min, after which 40 μL of 5 mM ferrozine (Sangon, Shanghai, China) was added to the solutions. After incubation at room temperature for 15 min, the absorbance at 562 nm was measured using a spectrophotometer. Each assay was performed in triplicate and the mean optical density value was used to calculate the percentage of Fe^2+^ deprivation. In addition, to determine whether the ferroxidase centers of rHrFer1 and rHrFer2 were active, the above assay was conducted under reducing conditions by the addition of Sn^2+^, a potent reducing agent of the Fe^3+^ that is possibly formed by the ferroxidase center. Briefly, after the first incubation, 20 μL of 0.16 M Sn^2+^ was added before the addition of ferrozine to the final reaction mixture.

### RNA interference

The data for the RNA interference (RNAi) system were obtained from the laboratory’s transcriptome database. The RNAi system was designed for the target sequences using si-Fi21 and BLOCK-iT RNAi Designer software (https://rnaidesigner.thermofisher.com/rnaiexpress/sort.do).

The double-stranded (ds) RNA-specific primers for the target genes were 5′-AGCCCCGTCAGAACTACCAT-3′ and 5'-GCCAGCTTGTGCAAGTCCAG-3' for the gene encoding HrFer1; 5'-GCACGCTTCTTCAGTGACCAGT-3' and 5'-CCAGAAGGAACTCGCCCAG-3' for the gene encoding HrFer2; and 5′-TAATACGACTCACTATAGGGACGTAAACGGCCACAAGT-3′ and 5′-TAATACGACTCACTATAGGGCTTCTCGTTGGGGTCTTT-3′ for the gene encoding the control, green flourescent protein (accession number U76561). The generated products were gel-purified and used to synthesize RNA through in vitro transcription with T7 RNA Polymerase (Roche), in accordance with the manufacturer’s instructions. To evaluate the efficiency of the RNAi, qPCR was carried out using SYBR Green.

Adult ticks were collected and divided into three groups: the control group, treatment group 1, and treatment group 2. Each group contained 60 adult ticks; 4000 ng dsRNA was injected into the fourth basal segment of each tick (the injection volume was calculated according to the concentration). After injection, the ticks were allowed to rest for 24 h in an incubator (25 °C, 95% relative humidity). They were then placed on the ears of rabbits for blood-feeding, and feeding duration, engorged body weight, egg weight and tick mortality calculated.

### Statistical analysis

The spatiotemporal expression of *HrFer1* and *HrFer2* was normalized according to that of β-actin and determined using the 2^−ΔΔCt^ method. The qPCR results were compared by one-way ANOVA using SPSS v17.0.

Student’s* t*-test (*P* < 0.05) was used to determine significant differences between the efficiency of the RNAi for each group, and one-way ANOVA (*P* < 0.05) for significant differences between engorged body weight, egg weight, tick mortality and feeding period of each group.

## Results

### Cloning and sequence analysis

The cDNA sequence of *HrFer1* contains a 519-base pair (bp) ORF, which potentially encodes 172 amino acids. The predicted molecular weight was 20 kDa and the isoelectric point 4.94 (Table 1). The complete cDNA sequence of *HrFer2* contains a 573-bp ORF, which potentially encodes 190 amino acids; the predicted molecular weight was 21 kDa and isoelectric point 5.51 (Table 1). The SignalP 4.1 server did not predict a signal peptide in *HrFer1*. The signal peptide of *HrFer2* was predicted at amino acids 1-15.

**Table 1:** The physicochemical parameters of *HrFer1* and *HrFer2*

Gene	ORF (bp)	Amino acids	Isoelectric point	Molecular weight (kDa)
*HrFer1*	519	172	4.94	19.865
*HrFer2*	573	190	5.51	21.496

*ORF* Open reading frame,* bp* base pairs

**Fig. 1:**
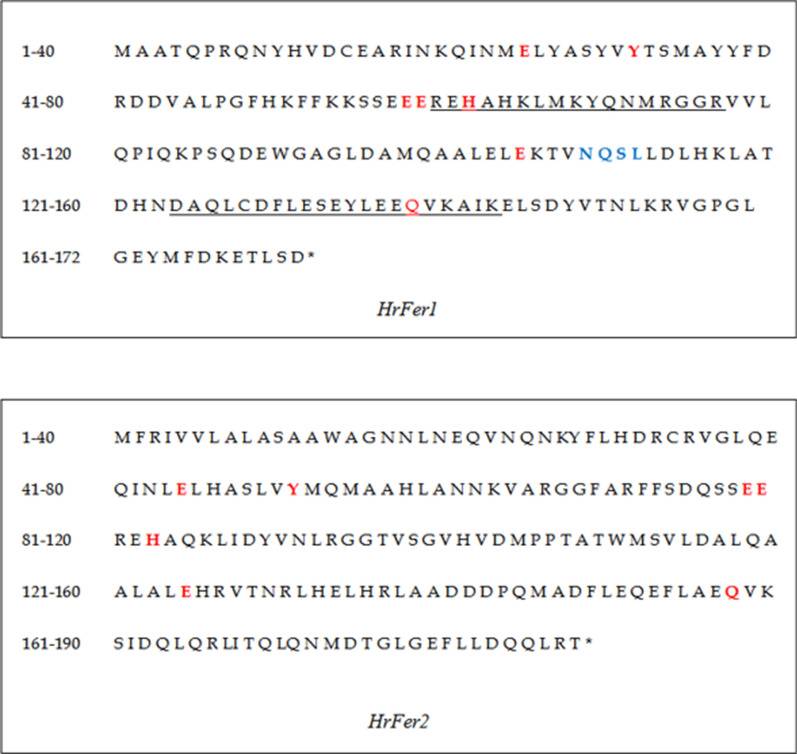
Amino acid sequences of *HrFer1 and HrFer2* [underlined sequences are the in-between ring fingers (IBR) domain S1 and IBRS2 of ferritin; seven conserved amino acids are marked in red and glycosylation sites are in blue]

The predicted amino acid sequences of *HrFer1* and *HrFer2* showed that they share seven amino acid residues unique to ferritin, namely Glu, Tyr, Glu, Glu, His, Glu, Gln. *HrFer1* contains two ferritin-specific domains and one glycosylation site, but *HrFer2* does not have these characteristics (Fig. 1).

**Fig. 2:**
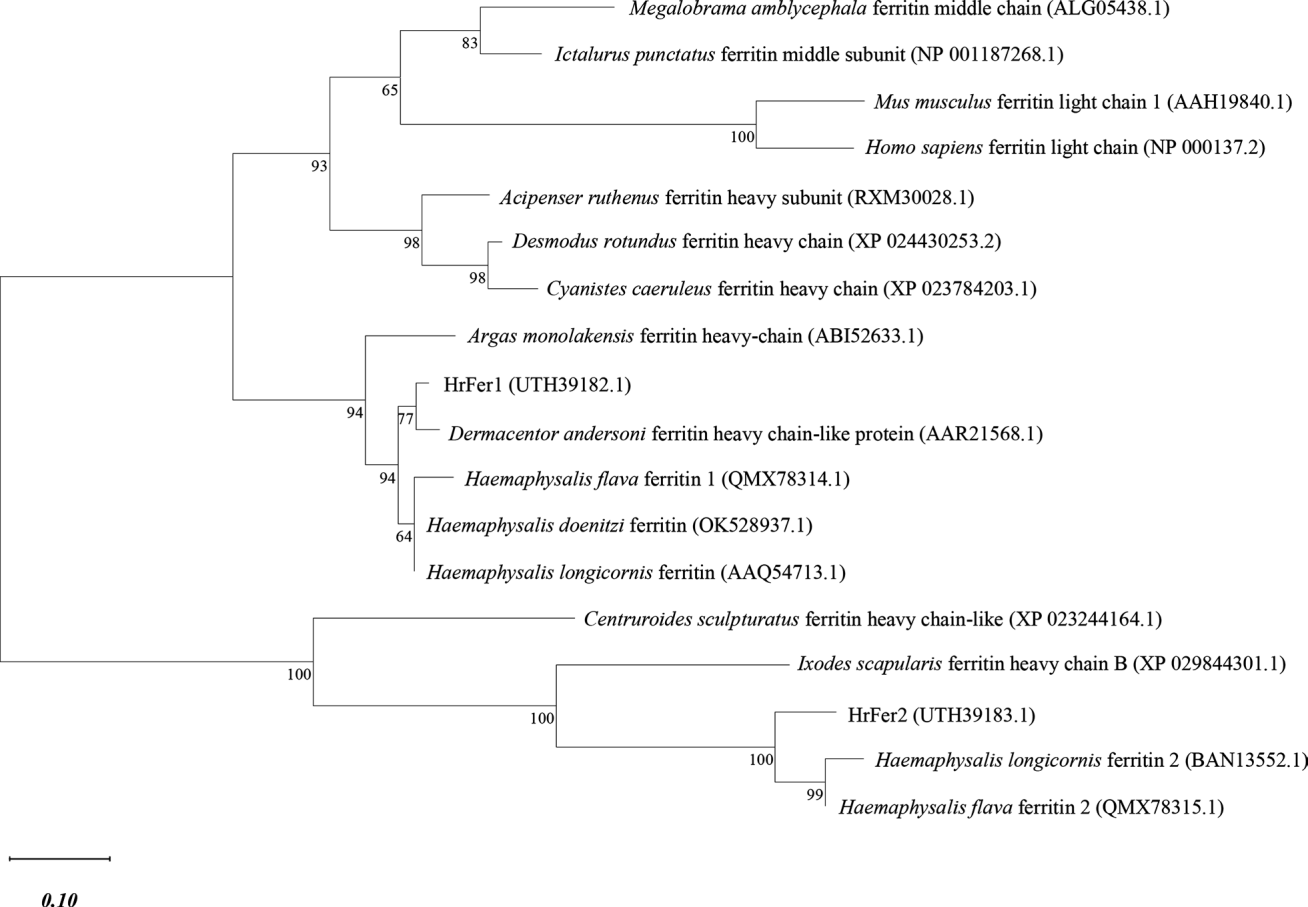
Phylogenetic tree showing the evolutionary relationships among different species based on the complete sequences of ferritins. The tree was constructed using maximum likelihood analysis of amino acid sequences of ferritin from 17 species. Bootstrap sampling was repeated 1000 times. The scale bar indicates the number of expected changes per site. Percentage bootstrap support is given at each node. GenBank accession numbers are listed after each species

The amino acid sequences of *HrFer1* and *HrFer2* of *H. rufipes* were compared with the ferritin sequences of different species from the NCBI database, and the following ferritin amino acid sequences with close homology were screened: *Ixodes scapularis* ferritin heavy chain B (XP_029844301.1), *Dermacentor andersoni* ferritin heavy chain-like protein (AAR21568.1), *Argas monolakensis* ferritin heavy-chain (ABI52633.1), *Haemaphysalis doenitzi* ferritin (OK528937), *Desmodus rotundus* ferritin heavy chain (XP_024430253.2), *Acipenser ruthenus* ferritin heavy subunit (RXM30028.1), *Cyanistes caeruleus* ferritin heavy chain (XP_0784203.1), *Centruroides sculpturatus* ferritin heavy chain-like (XP_023244164.1), *Megalobrama amblycephala* ferritin middle chain (ALG05438.1), *Ictalurus punctatus* ferritin middle subunit (NP_001187268.1), *Mus musculus* ferritin light chain 1 (AAH19840.1), *Homo sapiens* ferritin light chain (NP_000137.2), *Haemaphysalis longicornis* ferritin1 (BAN13552.1),* H. longicornis* ferritin2 (BAN13552.1), *Haemaphysalis flava* ferritin1 (QMX78314.1), *H. flava* ferritin2 (QMX78315.1). The ferritin genes of invertebrates and vertebrates were located in different branches. The light chain (L subunit), medium chain (M subunit) and heavy chain (H subunit) subunits of ferritin were also located in three different branches. *HrFer1* and *HrFer2* are located in different branches and have a distant evolutionary relationship. *HrFer1* and *HrFer2* are in the same branch as ferritin heavy chain subunit, so it is speculated that they are also ferritin heavy chain subunits (Fig. 2).

### Tissue distribution and expression

**Fig. 3:**
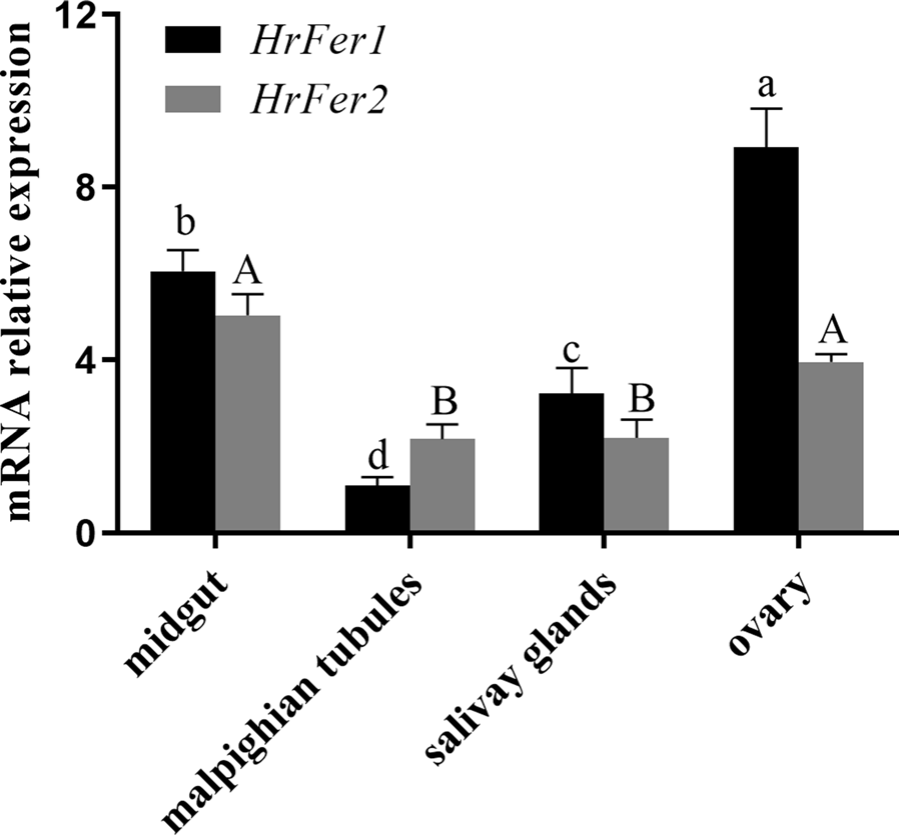
Relative expression of *HrFer1* and *HrFer2* in *Hyalomma rufipes* tissues assessed by Real time–quantitative PCR (RT-qPCR). The relative expression (fold) refers to *HrFer1/HrFer2* expression of adult ticks. The bars indicate the means and the error bars the SD. Different uppercase/lowercase letters indicate statistically significant differences (*P* < 0.05)

*HrFer1* was highly expressed in the ovary and midgut, followed by the salivary glands and Malpighian tubules. Its expression was significantly higher in the ovary and midgut than in the other tested tissues (df = 8, *P* < 0.05). *HrdFer2* was highly expressed in the midgut (df = 8, *P* < 0.05) (Fig. 3).

### Purification of rHrFer1 and rHrFer2

**Fig. 4:**
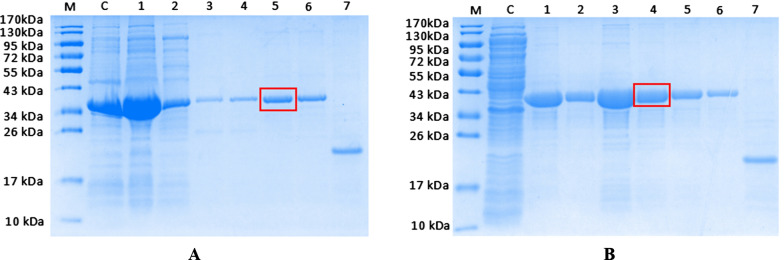
** a, b**Sodium dodecyl sulfate–polyacrylamide gel electrophoresis of His-tagged rHrFer1/rHrFer2 purified by nickel NTA affinity chromatography. Protein molecular weight marker (lane M); cell lysate excluding isopropyl β-d-1-thiogalactopyranoside (lane C); fraction washed from Ni–NTA with 20 mM imidazole (lane 1), 50 mM imidazole (lane 2), 100 mM imidazole (lane 3), 200 mM imidazole (lane 4), 250 mM imidazole (lane 5), and 500 mM imidazole (lane 6); Trx tag protein (lane 7)

The recombinant protein was purified by nickel column affinity chromatography and then eluted with different concentrations of imidazole. Purification was best at 250 mM imidazole for rHrFer1 (Fig. 4a) and at 200 mM imidazole for rHrFer2 (Fig. 4b).

### Mass spectrometric identification of rHrFer1 and rHrFer2

The recombinant plasmid was induced and expressed using 0.5 mM isopropyl-β-d-thiogalactoside at 37 ℃. Then, the correct target band identified by SDS-PAGE was cut out and enzymatic hydrolysis was carried out in the gel. Several specific peptides of ferritin were detected by liquid chromatography–tandem mass spectrometry, which proved that the recombinant protein was ferritin (Table 2).

**Table 2:** Mass spectrometric identification of rHrFer1 and rHrFer2

Protein	Unique peptides	Sequence	Theo MH+ [Da]	Proteins	Description	*r* (cross correlation)
rHrFer1	4	QNYHIDCEAR	1305.56406	1	Ferritin	2.13
		VGPGLGEYMFDK	1312.6242	1	Ferritin	2.29
		ELSDYVTNLKR	1337.70596	1	Ferritin	2.61
		TVNQSLLDLHK	1267.70048	1	Ferritin	2.76
rHrFer2	3	FFSDQSSEEREHAQK	1824.81473	1	Ferritin2	3.21
		LAADDDPQMADFLEQEFLAEQVK	2623.22322	1	Ferritin2	3.28
		LIDYVNLR	1005.57276	1	Ferritin2	2.12

### Iron chelating activity

**Fig. 5:**
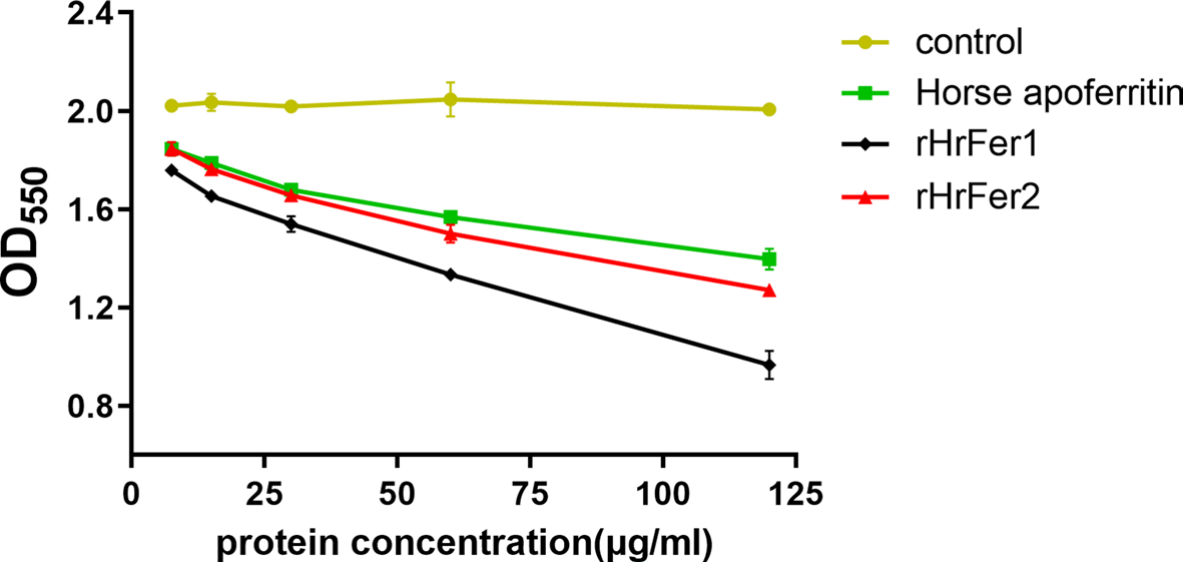
The iron chelating activity of rHrFer1 and rHrFer2 detected using the Fe^2+^ chelating reagent phenazine. Horse apoferritin was used as a positive control. rHrFer1 has stronger iron chelating ability than rHrFer2 under the same conditions. There was a significant difference between iron chelation by rHrFer1 and rHrFer2 and the control when the concentration of phenazine was above 60 μg/mL (df = 16, *P* < 0.05).* OD* Optical density

The iron chelating activity of rHrFer1 and rHrFer2 was detected using the Fe^2+^ chelating reagent phenazine. Both rHrFer1 and rHrFer2 showed concentration-dependent iron chelating activity, and there was a significant difference between their activity when the phenazine concentration was above 60 μg/mL (df = 16, *P* < 0.05) (Fig. 5).

### Effect of RNAi on biological characteristics of *H. rufipes*

**Fig. 6:**
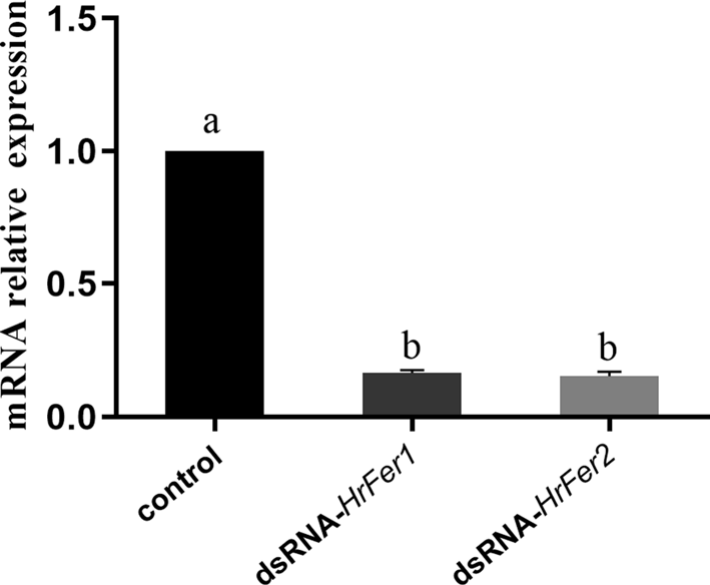
Relative messenger RNA (mRNA) expression of *HrFer1* and *HrFer2* in adult *Hyalomma rufipes* after RNA interference versus that of adult ticks microinjected with double-stranded RNA-targeting green fluorescent protein as a negative control. Each bar represents the mean and the error bars the SD. Different letters indicate statistically significant differences (df = 4, *P* < 0.05)

**Fig. 7:**
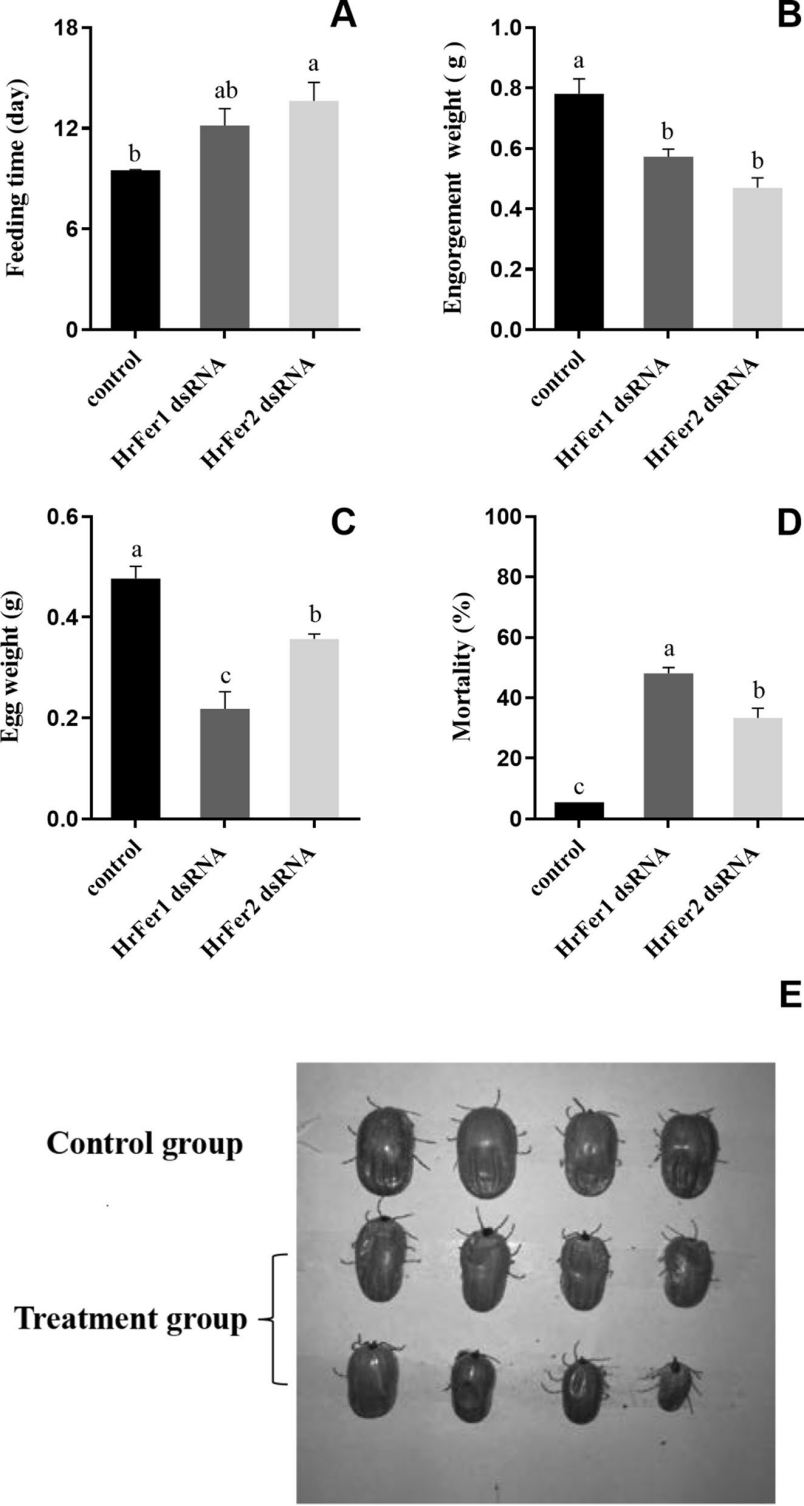
**a**–**e** Effects of RNA interference on the biological traits of *Hyalomma rufipes*. Each bar represents the mean and the error bars the SD. Double-stranded RNA-targeting green fluorescent protein was microinjected into *H. rufipes* as a negative control. **a** Time spent feeding, **b** engorgement weight, **c** egg weight, **d** mortality, **e** post-interference phenotype. Different letters indicate statistically significant differences (df = 6, *P* < 0.05)

After knocking down *HrFer1* and *HrFer2*, their relative expression decreased by 83.43% and 84.74%, respectively (Fig. 6). After knockdown of *HrFer1* and *HrFer2*, there were significant differences in feeding duration, engorged body weight, number of eggs laid and mortality between the treatment groups and the control group (Fig. 7). Engorged body weight and number of eggs laid decreased significantly after knockdown of *HrFer1* through RNAi, and mortality increased significantly (df = 6, *P* < 0.05). After knockdown of *HrFer2* through RNAi, feeding duration increased significantly (df = 6, *P* < 0.05), whereas engorged body weight and number of eggs laid decreased significantly (df = 6, *P* < 0.05); mortality also increased significantly (df = 6, *P* < 0.05). There was a significant difference in the number of eggs laid and mortality between the groups in which *HrFer1* or *HrFer2* were knocked down with RNAi (df = 6, *P* < 0.05); the number of eggs laid in the *HrFer1* knockdown group showed a more significant downward trend, and mortality in this group was higher.

### Effect of low temperature stress on the expression of *HrFer1* and *HrFer2*

**Fig. 8:**
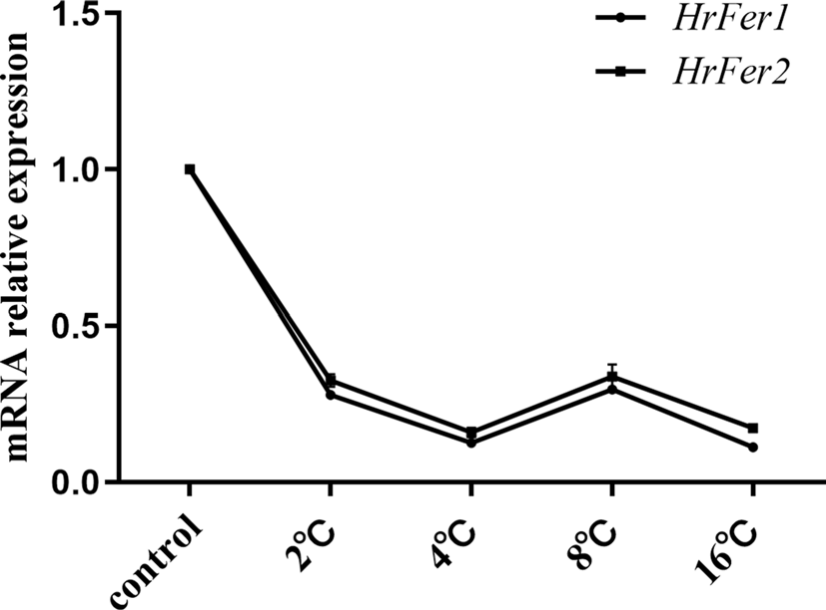
Effects of low temperatures on the expression of *HrFer1* and *HrFer2*. The relative expression (fold) refers to the *HrFer1/HrFer2* expression of an adult tick. The relative expression levels of *HrFer1* and *HrFer2* showed highly similar trends

Adult *H. rufipes* were subjected to low temperatures (2 ℃, 4 ℃, 8 ℃, 16 ℃) for 72 h, and then the relative expressions of *HrFer1* and *HrFer2* were determined. The relative expression of *HrFer1* and *HrFer2* showed a similar downward trend under low temperature stress (Fig. 8).

## Discussion

Ferritin, one of the major non-heme iron storage proteins in animals, plants and microorganisms, has many biological functions, including anti-oxidation, regulation of the metabolic balance of iron, and elimination of toxic heavy metals and other toxic molecules [9]. It is an iron-binding protein composed of 24 subunits that form a hollow spherical shell, and is involved in the transport and storage of iron during iron metabolism in most species of plants and animals [10–12].

The activity of recombinant ferritin proteins of *H. rufipes* was examined in the present study. The ferritins had good iron chelating activity, but that of rHrFer1 was stronger than that of rHrFer2. This difference may be related to the IBRS1 and IBRS2 domains of rHrFer1. However, it is worth noting that, because the recombinant protein is a fusion protein of Trx protein and HrFer, the conformation of the recombinant protein and the native protein is different. Due to technical limitations, there may have been some impurities in the recombinant proteins, which may have affected the accuracy of detection of their iron chelating activity. Thus, the iron chelating activity of HrFer2 may have been underestimated.

Common housekeeping genes used as reference genes for qPCR include β-actin, GAPDH, β-tubulin, GST and RPL4. Nijhof et al. [13] compared several housekeeping genes in *Rhipicephalus microplus*, and found that β-actin showed good stability. Furthermore, no significant change was found in the expression of β-actin at different development stages of *Hyalomma anatolicum*, which suggested its potential use as a reference gene [14]. β-actin was selected as the reference gene for the present study as it expressed stably in adult *H. rufipes*. However, its stability among different developmental stages of *H. rufipes* is not known, which limited its usefulness for evaluating the expression of specific genes at these stages.

Among the two subunits of vertebrate ferritin, the H subunit can oxidize toxic divalent iron ions in the body, and the L subunit can promote the mineralization and nucleation of trivalent iron ions [15]. Only the H subunit has thus far been found in tick ferritin. Homology between the H subunits of *HrFer1* and *HrFer2* and the M subunit of fish ferritin is higher than that with the L subunit of mammalian ferritin. In addition, H subunits of tick *HrFer1* and *HrFer2* belong to different branches, and it is speculated that there are some functional differences between them. The mechanisms of action of these two types of tick ferritin need to be further explored.

The ferritin heavy chain subunit of *H. rufipes* has an iron oxidase center, which is composed of seven conserved amino acid residues: Glu, Tyr, Glu, Glu, His, Glu and Gln. The light chain subunit has an iron nucleation site that can store Fe^3+^ and a salt bridge that stabilizes the protein structure, which is of great significance for the mineralization, nucleation and structural stability of ferritin [16].

Iron is an essential trace element for all living organisms. However, iron is potentially toxic due to its low solubility when its oxidation state is stable, i.e. Fe(III), and to its tendency to potentiate the production of reactive oxygen species [17]. Ticks take up a large amount of iron during a blood meal, and ferritin can regulate their iron balance. The active center of iron oxidase plays an important role in the process of binding Fe^2+^.

The relative expression of the ferritin genes was higher in the ovary and midgut of *H. rufipes*, and significantly different from that in the other tissues examined, indicating that they may have a more prominent function in the ovary and midgut. Ferritin1 of *Rhipicephalus sanguineus* plays a role in the development of oocytes [18]. There is high homology between *HrFer1* and ferritin1, and similarly to the expression of ferritin1 in *R. sanguineus*, the expression level of *HrFer1* was highest in the ovaries of *H. rufipes*. The transcription of ferritin2 was high in the *H. flavus* midgut [19]. Other arthropods, such as *Musca domestica*, also have higher levels of ferritin in their midguts [20].

In the present study, knockdown of *HrFer1* and *HrFer2* through RNAi had significant effects on engorged body weight, number of eggs laid and mortality of *H. rufipes*. In addition, knockdown of *HrFer2* by RNAi also significantly affected feeding duration of *H. rufipes*. Previous studies showed that the transcription of ferritins was upregulated in female ticks of *H. flavus* during blood-feeding, and the gene expression level of ferritin2 was much higher than that of ferritin1 in partially fed and engorged females [19]. The results of this latter study also confirmed that ferritins play an important role in blood-feeding in ticks, and that the mechanism of action is different for ferritin1 and 2. In the present study, *HrFer1* had a more significant effect on the number of eggs laid by *H. rufipes* than *HrFer2*. Silencing of ferritin1 in *H. longicornis *can directly cause the failure of egg-laying [21]. However, in the present study, knockdown of *HrFer1* only led to a reduction in the number of eggs laid by *H. rufipes*. Knockdown of ferritins in *H. flava* through RNAi led to a lower ratio of total weight of eggs laid to engorged body weight, and the eggs displayed abnormal morphologies [19]. In *Nilaparvata lugens*, silencing of ferritin1 heavy chain and ferritin2 light chain through RNAi led to undeveloped ovaries and severely inhibited oocyte growth [22]. In the present study, the expression of *HrFer2* was highest in the midgut of *H. rufipes*, which might explain the significant effect of *HrFer2* knockdown on the feeding duration of this tick. It has been confirmed that ferritin2 secreted in the midgut of *H. longicornis* [21] shows high homology with *HrFer2*.

RNAi has been widely used for the study of functional genomics in entomological research [23]. However, in ticks, RNAi is mainly applied with dsRNA microinjection. Due to the technical limitations of this method, there are only a few strategies that can be applied to overcome off-target effects. The three main strategies used to deal with off-target effects of RNAi are as follows: direct chemical modification of small interfering RNA (siRNA), using multiple siRNAs, and reducing the dose/concentration [24]. si-Fi21 software developed by Luck et al. [25] can be used for RNAi design and off-target prediction. This software optimizes the design process of RNAi to some extent, and also verifies that the silencing efficiency is positively correlated with the amount of effective siRNA [25]. BLOCK-iT RNAi Designer software, developed in accordance with Elbashir et al. [26], can be used to quickly and easily search for possible siRNA according to the target sequence. In the present study, bioinformatics software was used to analyze the target gene sequence, to design dsRNA sequence primers containing multiple siRNAs, and to conduct RNAi with relatively low concentrations of siRNAs under the same conditions. The optimal injection dose to reduce the off-target effect as much as possible was determined to be 4000 ng.

The relative expression level of ferritin heavy chain subunit from *Papilio xuthus* sharply increased in response to bacterial (*Escherichia coli* and *Staphylococcus aureus*) challenge [27]. The infection rate and proliferation rate of white spot syndrome virus in ferritin-silenced *Procambarus clarkii* were significantly increased [28]. In the present study, the relative expression of ferritin changed significantly under low temperature stress, and both *HrFer1* and *HrFer2* were significantly downregulated. It is interesting that their expression levels did not decrease when the treatment temperature decreased, and these results suggest that they maintained a relatively stable, low expression state under these conditions. Therefore, we speculate that the maintenance of physiological activities of ticks in low temperature environments may be related, to some extent, to ferritin. In other words, *HrFer1* and *HrFer2* may play a regulatory roles in the resistance of *H. rufipes* to low temperature stress.

## Conclusions

The activities of recombinant ferritin proteins of *H. rufipes HrFer1* and *HrFer2* were determined in the present study. The ferritin heavy chain subunits of *H. rufipes* have an iron oxidase active center. The relative expression of the ferritin genes was higher in the ovary and midgut of *H. rufipes*, and significantly different from those of the other tissues tested, which indicates that ferritin may have a more prominent function in the ovary and midgut of ticks. It was also found that *HrFer1* and *HrFer2* have an effect on blood meal digestion. Knockdown of *HrFer1* and *HrFer2* through RNAi had significant effects on *H. rufipes*, e.g. knockdown of *HrFer2* significantly affected the duration of blood-feeding in this tick. In sum, the results of this study illustrate some of the wide-ranging functions of ferritins in ticks, and help us to improve our understanding of the molecular basis of iron metabolism in these organisms.

## Data Availability

The complete sequences of *HrFer1* and *HrFer2* have been deposited in the NCBI Nucleotide database under the respective accession numbers UTH39182 and UTH39183. Inquiries for further information can be directed to the corresponding author.
